# Kinematic and electromyographic analysis of variations in Nordic hamstring exercise

**DOI:** 10.1371/journal.pone.0223437

**Published:** 2019-10-23

**Authors:** Nejc Šarabon, Jan Marušič, Goran Marković, Žiga Kozinc

**Affiliations:** 1 University of Primorska, Faculty of Health Sciences, Izola, Slovenia; 2 S2P, Science to practice, Ltd., Laboratory for Motor Control and Motor Behavior, Ljubljana, Slovenia; 3 University of Zagreb, Faculty of Kinesiology, Zagreb, Croatia; 4 Motus Melior Ltd., Zagreb, Croatia; 5 University of Primorska, Andrej Marušič Institute, Koper, Slovenia; University of Belgrade, SERBIA

## Abstract

The purpose of this study was to present and biomechanically evaluate several variations of the Nordic hamstring exercise (NHE), achieved by altering the slope of the lower leg support and by asumming different hip flexion angles. Electromyographic and 2D kinematic measurements were conducted to analyse muscle activity (biceps femoris, semitendinosus, gluteus maximus, erector spine and lateral head of the gastrocnemius), knee and hip joint torques during 6 variations of NHE. The study involved 18 adults (24.9 ± 3.7 years) with previous experience in resistance training, but with little or no experience with NHE. Increasing the slope of the lower leg support from 0° (standard NHE) to 20° and 40° enabled the participants to perform the exercise through a larger range of motion, while achieving similar peak knee and hip torques. Instructions for increased hip flexion from 0° (standard NHE) to 25°, 50° and 75° resulted in greater peak knee and hip torque, although the participants were not able to maintain the hip angle at 50° nor 75°. Muscle activity decreased or remained similar in all modified variations compared to the standard NHE for all measured muscles. Our results suggest that using the presented variations of NHE might contribute to optimization of hamstring injury prevention and rehabilitation programs, by providing appropriate difficulty for the individual’s strength level and also allow eccentric strengthening at longer hamstring lengths.

## Introduction

Nordic hamstring exercise (NHE) is commonly used in hamstring conditioning protocols, especially for injury prevention. Studies have shown that implementation of NHE into the training process can significantly reduce the incidence of hamstring strain injuries in high-speed running sports [1,2]. Moreover, numerous positive neuromuscular adaptations after performing the NHE have been demonstrated. For instance, significant improvement in eccentric hamstring strength was reported following an implementation of 4–10 weeks of NHE training [3–5]. Iga et al. [4] have also reported an improvement in eccentric hamstring strength at three angular velocities (60°/s, 120°/s and 240°/s) after a 4-week NHE intervention, even though the NHE was performed at a relatively slow pace. Improvements at higher angular velocities are likely one of the reasons why NHE is effective for hamstring strain injury prevention, considering that most hamstring strain injuries occur at high movement velocities (i.e., sprinting). Furthermore, significant lengthening effect on hamstring muscle fascicles resulting from an eccentric hamstring strengthening protocol have been shown [6–11]. Consequently, several research groups reported peak knee torque shifts towards position closer to complete knee extension (i.e., towards longer hamstring length) [5,8,12,13], which is another mechanism that is likely to contribute to decreased incidence of hamstring strain injuries, as these most often occur at longer hamstring lengths.

Although the effectiveness of NHE is well documented, several authors have pointed out that NHE has potential disadvantages. Brughelli and Cronin [14] have expressed doubt about whether NHE causes sufficient hamstring activity for optimal eccentric strengthening in its final phase (at smaller knee flexion angles). In addition, Ditroilo et al. [15] reported that the peak electromyographic (EMG) activity of hamstrings can be observed at 65.4 ± 8.4° knee flexion during the NHE and that a marked decrease is seen at 45° of knee flexion. Tillaar et al. [16] reported similar knee flexion angle at peak hamstring activation. They also showed an increase in hip flexion angle at the position of peak hamstring activity, which indicates difficulty to perform the NHE with an optimal form. Thus, the potential problem of NHE is the general difficulty of the exercise. Only sufficiently strong athletes are able to take full advantage of the exercise, with an active descend lasting until nearly fully extended knee position. Since the majority of the hamstring muscles also cross the hip in addition to the knee, performing NHE with neutral hip position does not enable strengthening the hamstrings at the longer lengths. This could be important drawback of NHE, since most of the hamstring strain injuries during sprinting [17] occur in the final part of the swing phase when the hamstring muscle-tendon complex reaches a significantly larger length compared to NHE, due to the significantly greater hip flexion (55–65°). [18] Therefore, the standard version of NHE might not be an optimal exercise for prevention of the hamstring strain injuries. Recently, attempts have been made to modify NHE in order to remove its drawbacks [19], however, the effects of different NHE variations on biomechanical parameters and muscle activity have not been thorougly tested yet.

The purpose of this study was to present and biomechanically evaluate potentially improved NHE variations that were obtained with (i) changing the slope of the lower leg support and (ii) changing the hip joint angle instructions. Such adjustments could possibly eliminate the existing disadvantages of the standard NHE. Including different variations of NHE in an individual’s training regimen could contribute to the larger or quicker hamstring muscle adaptations and further enhance its effectiveness in hamstring strain injury prevention. In our evaluation of different variations of NHE, we were primarily focused on differences in peak torques and angles at the knee and the hip joints, as well as peak EMG activity. We hypothesized that both approaches of NHE modifications (i.e. increasing the slope of the lower leg support and instructing to maintain larger hip flexion angle during NHE) would allow the participants to reach similar peak knee and hip joint torques at longer estimated hamstring lengths (reflected in larger knee and/or hip joint angles), compared to the standard NHE. Furthermore, we hypothesized that peak EMG activity of all measured muscles would also remain similar for all of the variations of NHE.

## Methods

### Participants

**Fig 1:**
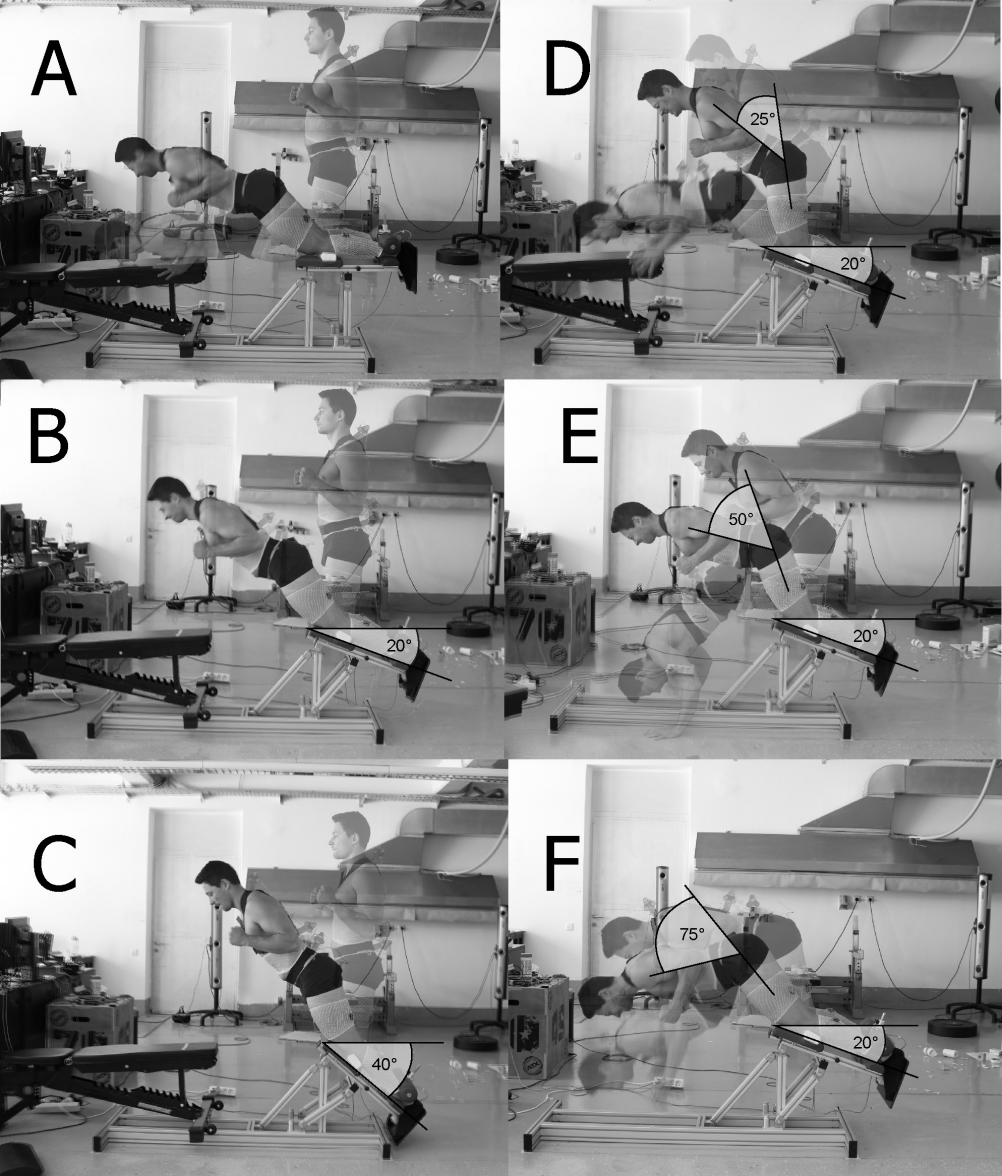
Depiction of NHE variations. Standard NHE (A) was modified by changing the slope of the lower leg support (B– 20°, C– 40°) and by instructing the participants to maintain different hip flexion angles throughout the movement (D– 25°, E– 50°, F– 75°).

**Fig 2:**
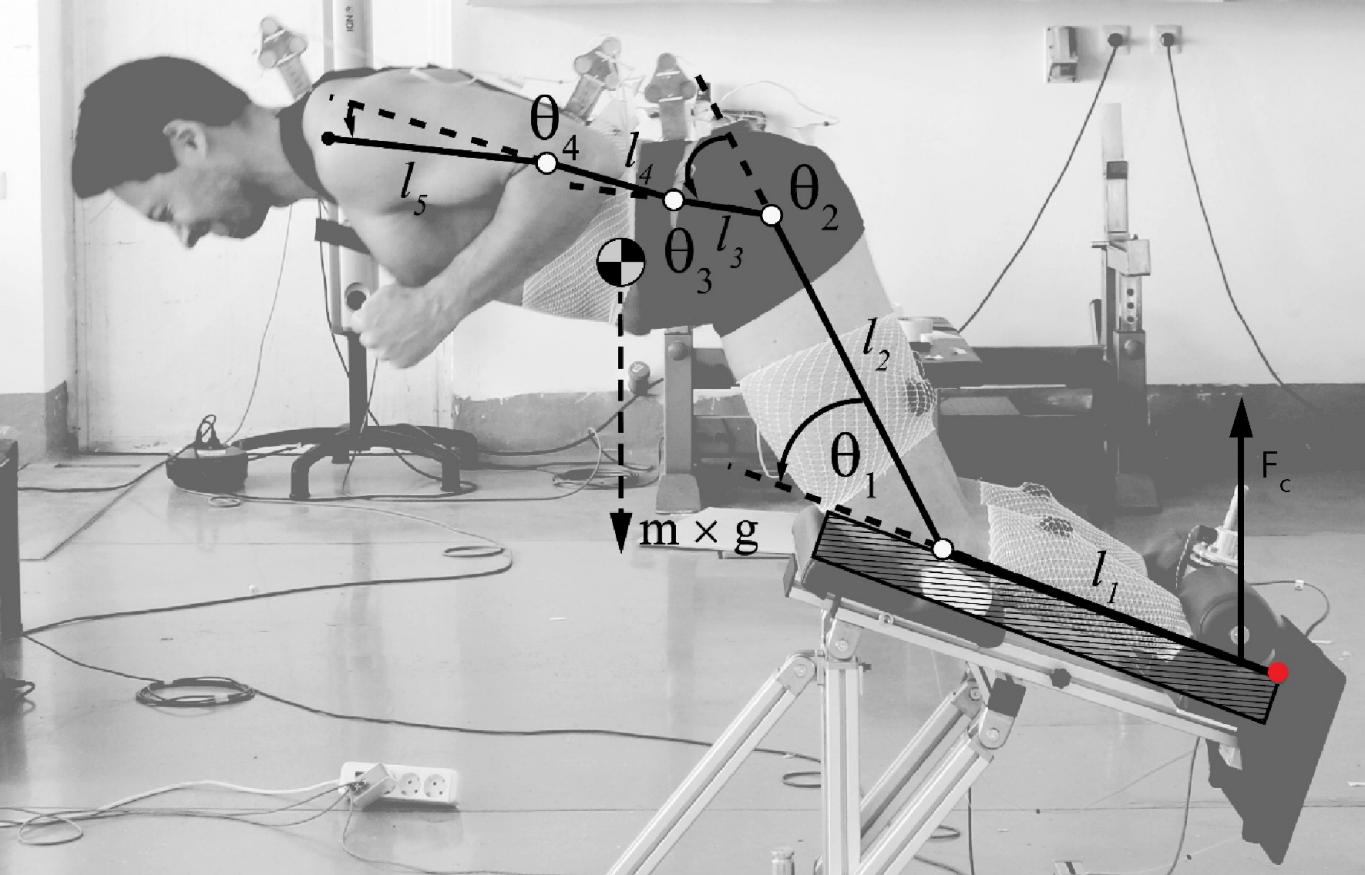
Planar inverse dynamics model. The participant performing the task motion was represented by a five-segment model. Segment angles for the shank (*l*_*1*_), thigh (*l*_*2*_), pelvis (*l*_*3*_), lumbar spine (*l*_*4*_) and thoracic spine (*l*_*5*_) were defined as shown. The origin of the model, which is also the point where the model is fixed to the base, is marked with a red dot. F_c_ (contact force) was measured by the built-in dynamometer.

Eighteen healthy volunteers (5 females, 13 males) participated in the study. The sample characteristics were (mean ± SD): age 24.9 ± 3.7 years, body mass 74.1 ± 14.1 kg, body height 176.0 ± 8.9 cm, BMI 23.7 ± 2.6 kg/m^2^, body fat 15.9 ± 4.3%, muscle mass 79.9 ± 4.2%. Minimal sample size of 15 participants was determined a priori for 80% statistical power, an alpha error of 0.05 and an effect size of 0.5. The inclusion criteria were: performing regular physical activity, experience with strength training, little or no experience with NHE and the ability to descend actively to at least 50% of the range of motion in the standard NHE. The exclusion criteria were: neural, muscular, skeletal or connective tissue injuries during the last 12 months in the area of the back, hips and legs. All participants were informed about the purpose and content of the study and gave written informed consent prior to participation. The individual pictured in Fig 1 and Fig 2 has provided written informed consent (as outlined in PLOS consent form) to publish their image alongside the manuscript. The study was approved by the National Medical Ethics Committee (0120-690/2017/8) and conducted according to the Declaration of Helsinki.

### Study protocol

To assess biomechanical differences between NHE variations, a single-visit cross-sectional study design was used. Before the warm-up, body mass, body fat percentage and muscle mass percentage were measured using a bio-impedance scale (Tanita MC-980MA, Tanita, Tokyo, Japan). Then, the participants performed a warm-up, consisting of light aerobic activity (6 minutes of alternating stepping on a 25 cm high box), 8 repetitions of dynamic stretching exercises (hip circles, forward, backward and side hip bending, leg swings) and 10 repetitions of strength bodyweight exercises (squats, heel rises, hip bridges, Jackknife sit-ups and hip extensions). After the warm-up, EMG electrodes and kinematic markers were attached (detailed locations are described in further paragraphs). Five repetitions of each variation were performed, with 2 additional familiarization repetitions before each NHE variation. The rest between NHE variations was 3 minutes while the rest between familiarization and actual trials of the same variations was 2 minutes. The rest between each repetition within each NHE variation was sufficent for the subject to comfortably return into the starting position (5–10 seconds). The order of the variations was randomized between participants. After performing all NHE variations, the kinematic markers were removed, and participants performed maximal voluntary isometric contractions (MVC) for the purpose of EMG normalization. For each muscle, 3 repetitions of 3-second maximal isometric exertion against external resistance were performed as follows: trunk extension in isometric dynamometer (S2P, Science to practice, Ltd., Ljubljana, Slovenia) in an upright stance with fixed pelvis for the erector spine muscles, hip extension in a prone position on a physio bed against fixed straps, placed just above the knee, with 90° knee flexion for gluteus maximus, plantar flexion against external resistance in neutral ankle position in an upright stance (attempting to lift overloaded Olympic bar on a smith machine, using only ankle joint) for the lateral head of gastrocnemius and knee flexion in a prone position on a physio bed against fixed straps, placed above the calcaneus bone, with knee flexed at 45° for the biceps femoris and semitendinosus. The knee angle was determined in view of the previous studies [20–22], showing highest knee flexion torque during isokinetic tests and a similar EMG activity of the biceps femoris and semitendinosus at 45° of knee flexion. Loud verbal encouragement by the examiner was provided during all MVC trials.

### Nordic hamstring exercise variations

For the implementation of all variations of NHE, a custom-designed device with adjustable length and slope of the lower leg support was used (S2P, Science to practice, Ltd., Ljubljana, Slovenia). Table 1 and Fig 1 show key differences between NHE variations used in this study.

**Table 1:** Key differences between NHE variations.

Variations in Fig 1	Slope of the lower leg support	Hip flexion instruction
A	0°	0°
B	20°	0°
C	40°	0°
D	20°	25°
E	20°	50°
F	20°	75°

Three different slopes (0°, 20°, 40°) of lower leg support and four hip flexion positions (0°, 25°, 50° and 75°) were used. For all variations with different hip angle instructions, the slope of the lower leg support was set at 20°. Note that the amount of hip flexion refers to researcher’s instruction and was not necessarily maintained by the participants to the end of the range of motion. Before each repetition, the appropriate hip flexion was determined using a goniometer. Participants were instructed to position the hands along the body, with an approximately 130° elbow flexion in all variations (Fig 1).

### Data acquisition and processing

Contact forces were measured at a sampling rate of 500 Hz at the ankle support (Fig 2) using a built-in dynamometer (Optoforce 3D, Budapest, Hungary). Three-dimensional marker trajectory data were collected at a sampling rate of 100 Hz, using the 3D Optotrak Certus motion capture system with 2 cameras (NDI Inc., Ontario, Canada). Active markers were placed (unilaterally) on the bony landmarks of at the ankle (lateral malleoli), knee (lateral condyle of tibia) and hip (greater trochanter). Additionally, five rigid marker clusters were secured to the pelvis (cluster at the sacrum), lumbar region (cluster near the thoracic level of T12), upper body (cluster near the cervical level of C7) and mid-upper arm and mid-lower arm. Small gaps of missing data were filled in using linear interpolation method. Signals were smoothed using low frequency fourth-order Butterworth filter, with 5 Hz cut-off frequency [23]. Joint torques were calculated by using a planar (2D) inverse dynamics model, built with a segmental method [24,25] and by using the segments’ inertial parameters from de Leva [26]. The model consisted of 5 segments (shank, thigh, pelvis, lumbar and thoracic spine) and 4 joints (knee–q1, hip–q2, lumbar–q3 and thoracic–q4) and it was fixed to the base at the beginnig of the first segment (Fig 2). 2D joint moments were then computed in MATLAB 2015b (The MathWorks, Natick, USA) in which we used the Spatial_v2 package as by Featherstone [27]. Main outcome measures were peak knee torque, peak hip torque (not necessarily achieved at the same time point during the exercise) and peak hip + knee torque (the higest sum of the torques at the same time point). Additionally, we calculated knee, hip, hip + knee and lumbo-pelvic angles at time point of peak hip + knee torque.

For EMG activity assessment, Trigno Delsys Wireless System was used (Delsys Inc., Massachusetts, USA), with pre-amplified self-adhesive wireless electrodes (dimensions: 27 x 37 x 15 mm; mass: 14.7 g; electrode material: silver; contact dimension: 5 x 1 mm) placed bilaterally on erector spine muscles, gluteus maximus, biceps femoris, semitendinosus and lateral head of the gastrocnemius. Prior to sensor placement, the skin over the muscles was shaved, abraded and cleaned with alcohol. EMG sensors were placed according to SENIAM recommendations [28], as shown in Table 2. Their location was confirmed with palpation and isometric muscle contractions.

**Table 2:** Locations and orientations of electromyographic sensors for different muscles, as recommended by SENIAM [28].

Muscle	Location	Orientation
Erector Spinae	At 2 finger width lateral from the proc. spin. of L1.	Vertical.
Gluteus Maximus	At 50% on the line between the sacral vertebrae and the greater trochanter.	In the direction of the line from the posterior superior iliac spine to the middle of the posterior aspect of the thigh.
Biceps Femoris	At 50% on the line between the ischial tuberosity and the lateral epicondyle of the tibia.	In the direction of the line between the ischial tuberosity and the lateral epicondyle of the tibia.
Semitendinosus	At 50% on the line between the ischial tuberosity and the medial epycondyle of the tibia.	In the direction of the line between the ischial tuberosity and the medial epycondyle of the tibia.
Lateral head of the gastrocnemius	At 30% on the line between the head of the fibula and the heel.	In the direction of the line between the head of the fibula and the heel.

The EMG data was acquired at 2000 Hz and processed in the following order: 1) band pass filtration using Butterworth second-order filter (20–500 Hz), 2) rectification, using root mean square function (0.05 second window length and point-by-point overlap), 3) smoothing, using moving average function (0.05 second window length and point-by-point overlap). The main outcome measure was peak EMG activity for all muscles, which was determined as highest mean value on 0.25 window length and expressed as percentage of maximal EMG activity during MVC trials (processed in the same order and calculated as maximal value on a 0.25 window length, which is in line with previous studies) [29].

### Statistical analysis

The data were statistically processed in the SPSS 22 computer program (IBM, New York, USA). Descriptive statistics were calculated and reported as mean ± standard deviation. Shapiro-Wilk test was used for testing of normality and Levene’s test for equality of variances. Differences among corresponding variables obtained from different NHE variations were tested with the analysis of the variance for repeated measurements. For pair-wise comparisons, paired 2-tailed post-hoc t-tests with Bonferroni’s correction were used. Furthermore, the effect sizes were calculated (Cohen's d) and interpreted as small (d = 0.2), moderate (d = 0.5) and large (d = 0.8) [30]. The level of statistical significance was set at p < 0.05 for all analyses.

## Results

**Fig 3:**
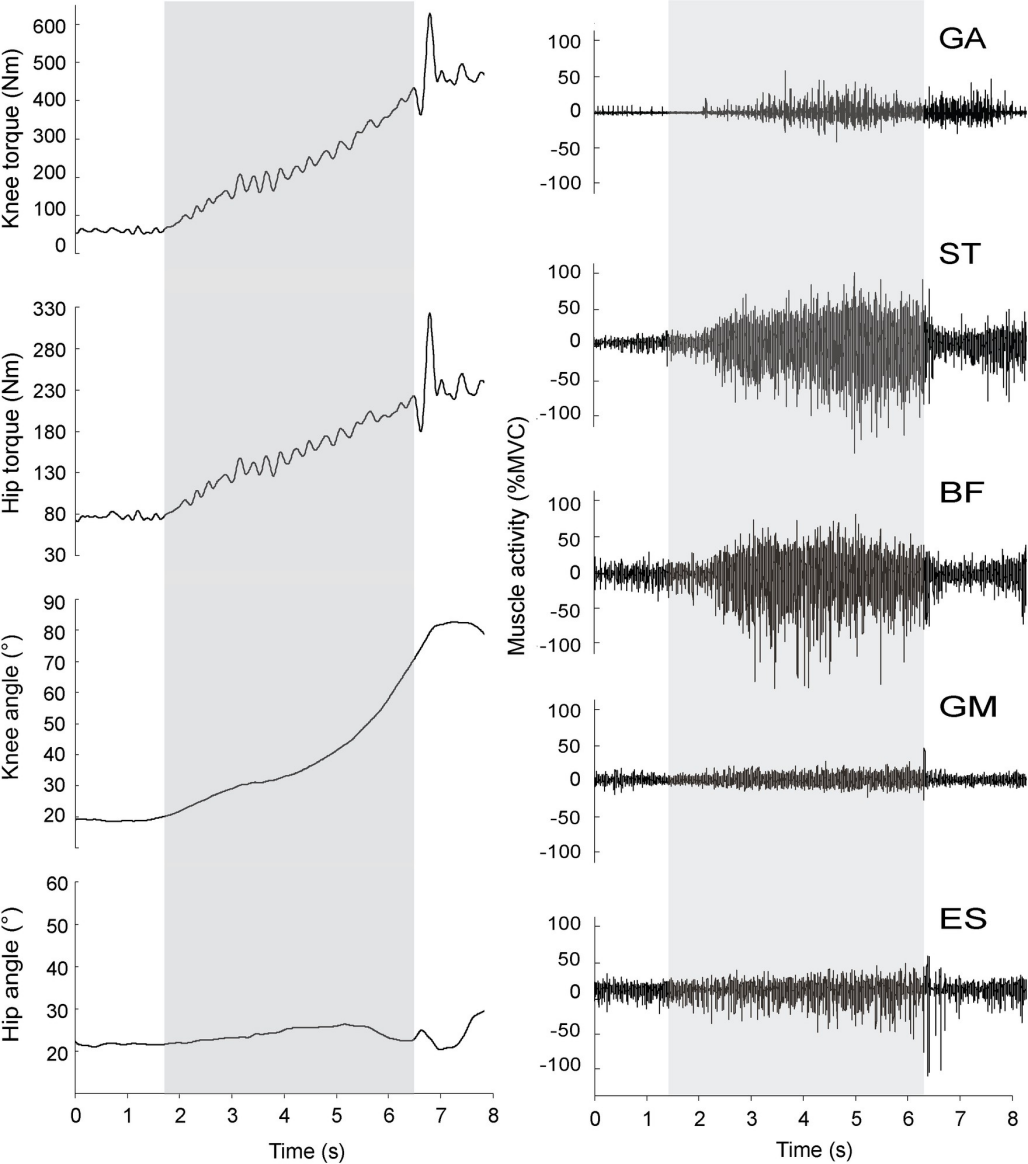
A representation of a typical kinematic values and raw EMG signals. Data is presented for one repetition during the variation with 20° slope of lower leg support and instructions to maintain 25° of hip flexion. The grey area represents the analyzed timeframe, after which the participants ceased to maximally contract the muscles and dropped down. Signals were manually inspected and time of actual peak torques were determined. GA–gastrocnemius, ST–semitendinosus, BF–biceps femoris, GM–gluteus maximus, ES–erector spinae.

All participants performed all variations of the NHE. An example of joint torque, joint angles and raw EMG activity signals for one repetition is shown in Fig 3.

### Peak joint torques

**Fig 4:**
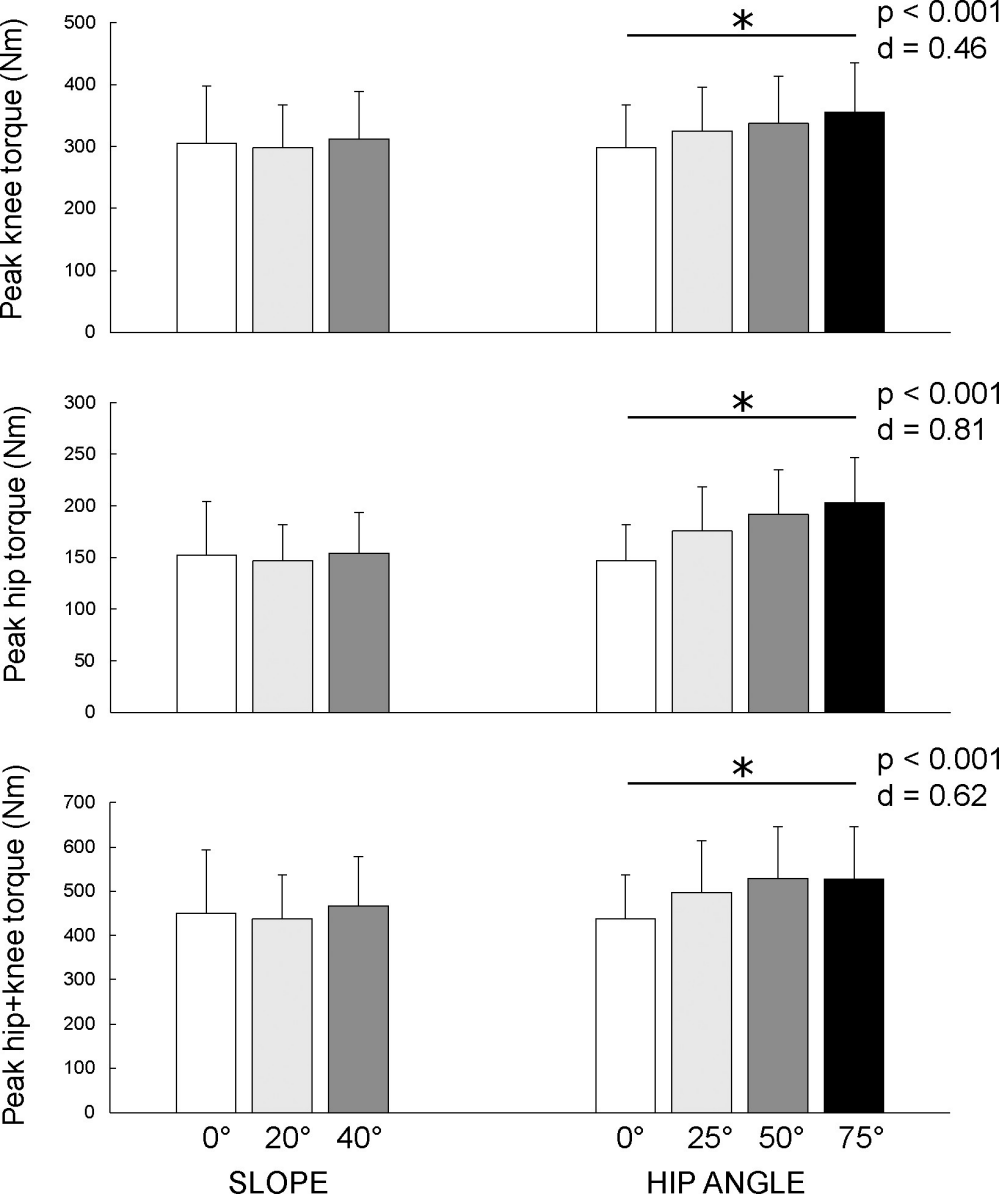
Comparison of peak knee, peak hip and peak hip + knee torque. NHE variations were performed with 3 different slopes of the lower leg support (0°, 20°, 40°; all variations with 0° hip flexion) and 4 different hip flexion angle instructions (0°, 25°, 50°, 75°; all variations with 20° slope). Asterisks indicate significant differences across all hip angles variations.

Changing the slope of the lower leg support did not affect peak torque at the knee (F_(2)_ = 0.651; p = 0.528; d = 0.037), hip (F_(2)_ = 0.607; p = 0.551; d = 0.034) or hip + knee (F_(2)_ = 1.073; p = 0.353; d = 0.059). Instructing the participants to maintain different hip flexion angles had a statistically significant effect on peak knee, hip and hip + knee torques (F = 15.008–74.101; all p < 0.001, d = 0.496–0.813). Lowest peak knee torque was achieved in 0° hip flexion variation (297.69 ± 69.98 Nm) and highest in 75° hip flexion variation (355.54 ± 78.82 Nm). Similar trend was observed for hip and hip + knee peak torques. Changes in peak torque data are shown on Fig 4.

### Joint angles

**Fig 5:**
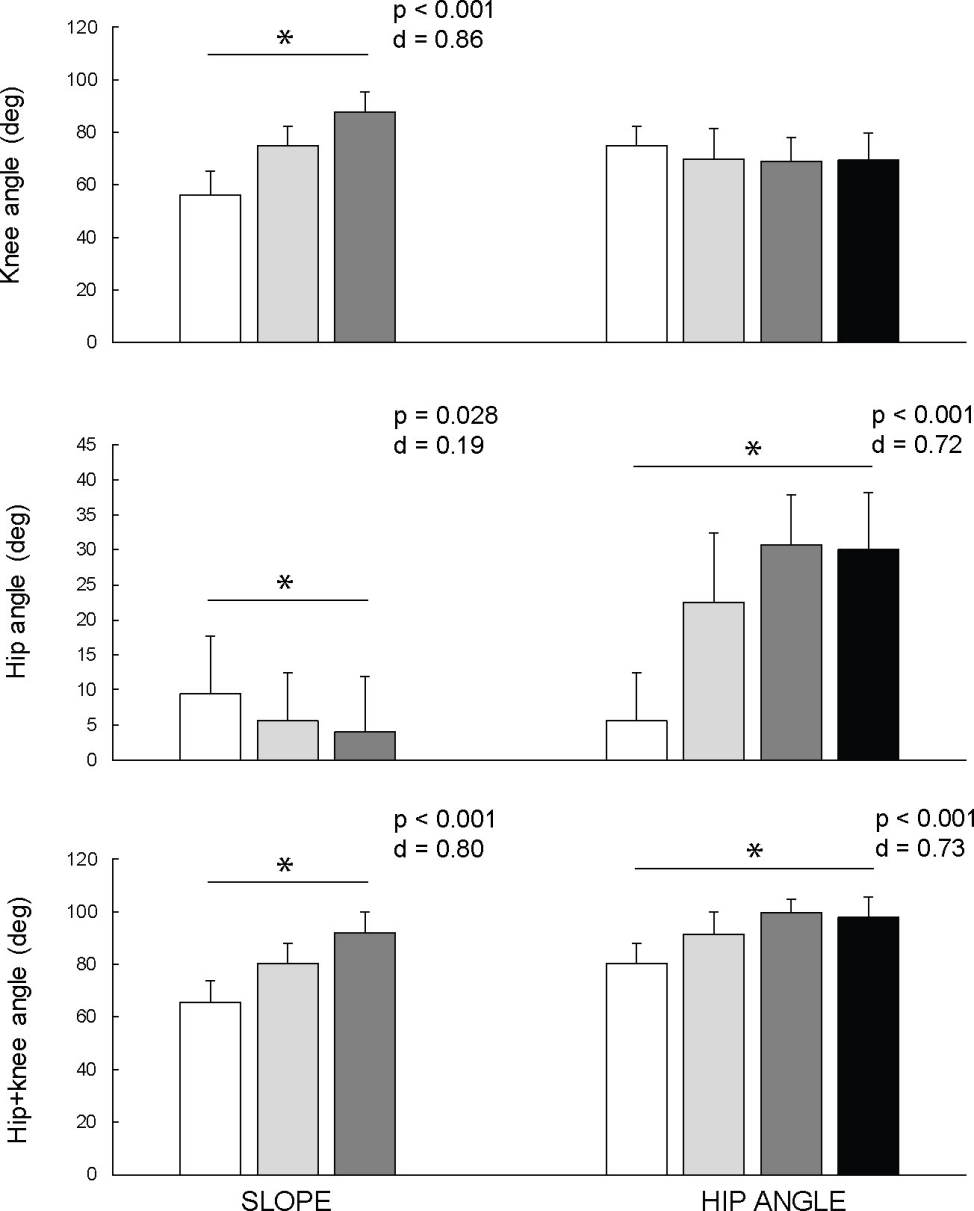
Comparison of joint angles at the time point of peak hip + knee torque. Nordic hamstring exercise variations were performed with 3 different slopes of the lower leg support (0°, 20°, 40°; all variations with 0° hip flexion) and 4 different hip flexion angle instructions (0°, 25°, 50°, 75°; all variations with 20° slope). 0° knee angle represents knee angle at the starting position of NHE. 0° hip angle represents neutral hip position. The sum of hip and knee angle represents estimated hamstring length. Asterisks indicate significant differences across all hip angles variations.

Joint angles were analysed at the time point of peak hip + knee torque. Note that 0° represents knee angle at the starting position of NHE and thus it increases during the descend towards the full knee extension. This method was used to enable for calculation of the estimated hamstring length, i.e. hip + knee angle, in which greater values represents longer hamstring length. Knee angle at the moment of peak hip+knee torque was significantly increased in NHE with 20° slope (75.01 ± 7.30°) and 40° slope (87.91 ± 7.45°) of lower leg support, compared to the standard NHE (56.10 ± 9.08°) (F_(2)_ = 100.3; p < 0.001; d = 0.855). Knee angle remained similar as changing hip flexion instructions (F_(3)_ = 2.510; p = 0.069; d = 0.129). Hip flexion angle at the moment of peak hip+knee torque was significantly decreased in NHE with 20° slope (5.64 ± 6.76°) and 40° slope (3.94 ± 8.06°) of lower leg support, compared to the standard NHE (9.39 ± 8.36°) (F_(2)_ = 67.31; p < 0.001; d = 0.798). Hip flexion angle at the moment of peak hip+knee torque was significantly increased at larger hip flexion instructions (F_(2)_ = 46.23; p < 0.001; d = 0.731). However, no differences were shown by pairwise tests between 50° and 75° variation (t_(17)_ = 1.61; p = 0.125; d = 0.133). Participants did not maintain the instructed hip flexion angles in 50° and 75° variations at the moment of the peak torque (26.94 ± 9.46° and 25.23 ± 16.62°, respectively). Lumbar-pelvic angle at the moment of peak hip+knee torque increased with increasing angle of hip flexion instructions (F_(3)_ = 28.08; p < 0.001; d = 0.623), but not with changing the slope of lower leg support (F_(2)_ = 2.76; p = 0.077; d = 0.140). Changes in joint angles at the time point of peak hip + knee torque are shown on Fig 5.

### Muscle activity

**Fig 6:**
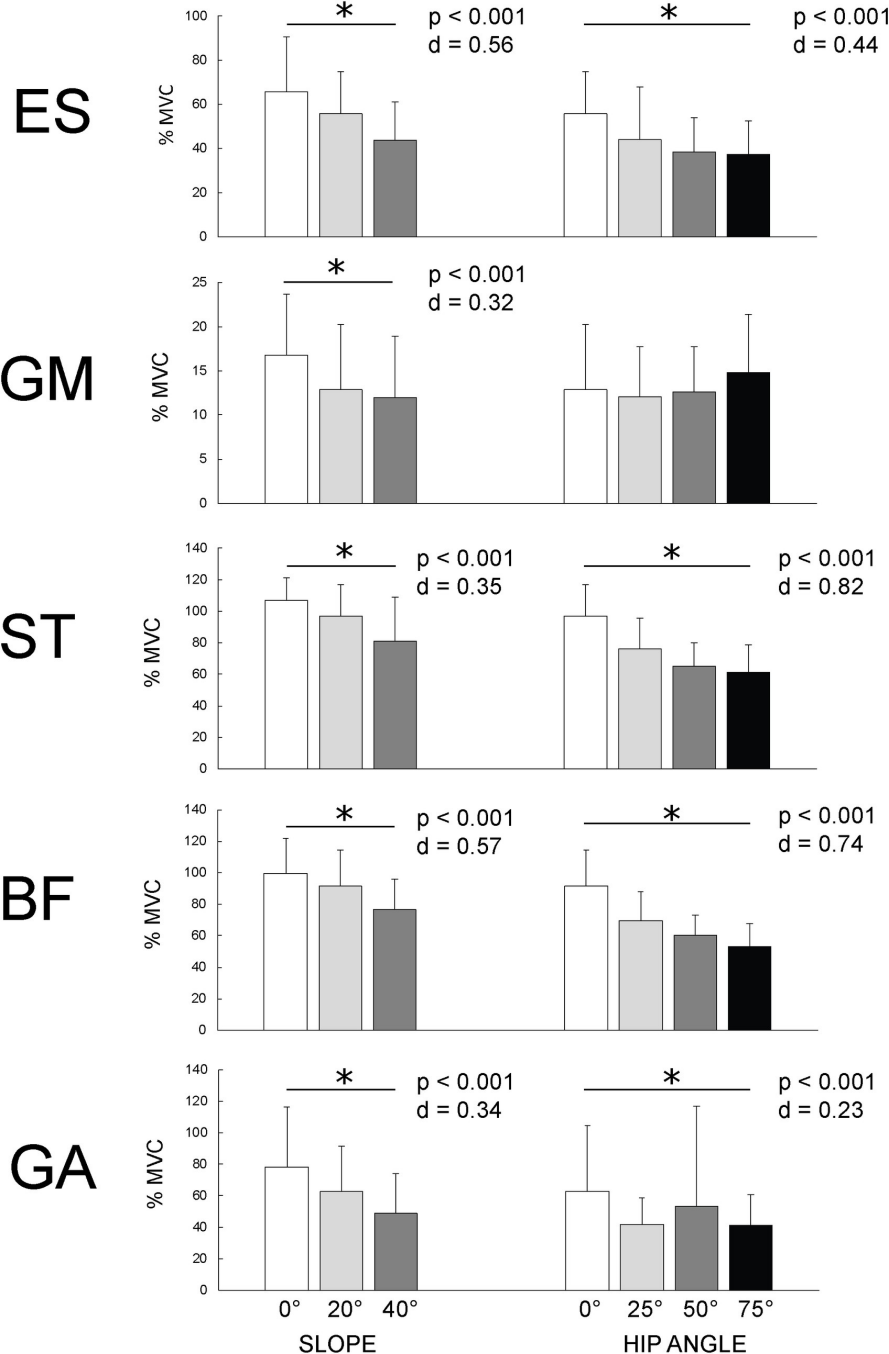
Comparison of EMG activity. Measured muscles were: GA (lateral head of the gastrocnemius), BF (biceps femoris), ST (semitendinosus), GM (gluteus maximus) and ES (erector spinae) in 3 different slopes of the lower leg support (0°, 20°, 40°; all variations with 0° hip flexion) and 4 different hip flexion angle instructions (0°, 25°, 50°, 75°; all variations with 20° slope).

For all measured muscles in all NHE variations, peak EMG activity was detected at the moment (± 25 ms) of peak hip + knee torque. Increasing the slope of lower leg support significantly decreased EMG activity of all analyzed muscles (F_(2)_ = 8.36–22.29; p = 0.001-0.002; d = 0.343–0.567) (Fig 5). Pairwise comparisons revealed statistically significant differences between 0° and 20° slopes for all muscles except semitendinosus (p = 0.06) and between 20° and 40° slopes for all muscles except gluteus maximus (p = 0.495). Changing the instructed hip flexion position decreased EMG activity of all muscles (F_(3)_ = 4.58–79.15; p = 0.000-0.007; d = 0,223-0,744), except gluteus maximus (p = 0.287). With the exception of gastrocnemius, there was a trend for EMG activity to drop with increasing hip flexion angle. Changes in peak EMG activity are shown on Fig 6.

## Discussion

The main purpose of this study was to evaluate biomechanical differences between the six different NHE variations. We hypothesized that the increased slope of the lower leg support and instructions to maintain larger hip flexion would allow the participants to perform the exercise through greater amplitude and reach peak joint torques at longer hamstring lengths, compared to the standard NHE. The results confirm that modifying the slope of the lower leg support allowed the participants to perform the movement through greater amplitude in controlled manner, since the peak joint torques occurred closer to the full knee extension (Fig 5). Morever, compared to the standard NHE, peak joint torques were reached at longer hamstring lengths in all modified variations (Fig 5). The level of the peak torque remained similar in the variations with different inclination of the lower leg support or was increased in the variations with the instruction to sustain hip flexion position (Fig 4). Despite that, peak EMG activity of all measured muscles significantly decreased in all modified varations, compared to the standard NHE. Furthermore, the participants in our study were only able to maintain small to modrate hip flexion throughout the exercise (Fig 5), indicating that our results demonstrate only the effect of different instructions to participants, not the effect of the actual hip flexion angle. Variations with instructions to maintain 50° and 75° of hip flexion turned out to be difficult to perform. In particular, the participants often substantially flexed the lumbar spine, despite being instructed to maintain neutral spine position.

### Peak joint torques and joint angles

The rationale behind changing the slope of the lower leg support was to decrease the knee flexion angle at the moment of peak knee torque, in order to allow an individual to perform NHE through greater range of motion in a controlled manner. Furthermore, performing the exercise with flexed hips could additionally lengthen the hamstring muscles. That would enable eccentric strengthening at hamstring lengths similar to those at which most strain injuries occur during sprinting. Our results show that all modified variations of NHE allowed the participants to reach peak hip and knee torques at longer estimated hamstring lengths (Fig 5). Specifically, during the standard NHE, similar peak joint torques occurred at a smaller knee angle (56.10 ± 9.08°), compared to the modified variations, during which participants could achieve nearly complete knee extension (75.01 ± 7.30° at 20° slope and 87.91 ± 7.45° at 40° slope). Moreover, when participants were instructed to maintain larger hip flexion angles, their average estimated hamstring length (hip + knee angle) at the moment of peak torques increased, mostly as a result of increased hip angle (Fig 5). At the same time, this type of modification of NHE proved to be effective for increasing peak knee torque, which is in line with the results of previous research that reported larger knee flexion strength when the hip is flexed [20,31]. It is known that training at longer hamstring lengths is effective for hamstring strain injury rehabilitation [32] and can favorably affect several architectural and functional characteristics of the hamstring muscles, but the underlying mechanisms are not yet completely understood. Recently, Guex et al. [8] compared eccentric hamstring conditioning protocols at shorter and longer lengths, and reported no significant differences in changes in fascicle length nor pennation angle. Although we demonstrated favorable effects of NHE variations on kinematic variables, further studies are needed to confirm whether performance of these variations lead to different architectural and functional adaptations of the hamstring muscles, compared to the standard NHE.

It is necessary to stress the discrepancy between the instructions for keeping a particular hip flexion angle (0°, 25°, 50°, 75°) during the NHE and the hip flexion value that was actually maintained at the moment of peak hip + knee torque. Namely, the average hip flexion achieved at the moment of the peak hip + knee torque during variations with the instruction for 50° and 75° hip flexion were only 30.74 ± 7.10° and 29.99 ± 8.19°, respectively. Kinematic data also shows that the average hip flexion during 50° and 75° NHE variations was already 10–20° lower than instructed in the starting position (despite using the goniometer and constant verbal warnings) and that it rapidly declined in the last 20% of the range of motion. In contrast, lumbar-pelvic angles significantly increased with increased level of the instructed hip flexion, which implies that the participants did not manage to maintain neutral lumbar curvature as instructed. By attempting to follow the instructions for larger hip flexion (50° and 75°), the participants actually performed a combination of hip flexion, pelvic rotation and spine flexion. Future studies are needed to define what level of NHE strength is needed to perform these variations of NHE without lumbar flexion. Based on these results and the fact that a big proportion of high-level athletes and most of the recreational athletes are not able to perform the standard NHE through full range of motion [4,15], we propose that those individuals start with with neutral hip position on either 20° or 40° slope of the lower leg support (depending on their strength level). The next step in progression would be gradually increasing hip flexion, while lowering the slope of the lower leg support to 0°. When an individual is capable to perform the NHE at the 0° slope of the lower leg support and with 75° of hip flexion, he/she can then progress to the weighted variations.

### Muscle activity

Despite the fact that the peak knee and hip torques remained similar (in variations with increased lower leg support slope) or were increased (in variations with increased hip flexion angle) compared to the standard NHE, the peak hamstring activity significantly decreased during all modified NHE variations. Moreover, the largest peaks in knee and hip torque were achieved concomitantly with the lowest peak hamstring EMG activity. A possible explanation for this phenomenon is that the non-contractile elements contributed a larger proportion of the force, due to the longer length of the hamstrings. The results are consistent with the findings of Higashihara et al. [22] and Lunnen et al. [33], who reported inverse relationship between hamstring EMG activity and hamstring length during eccentric and isometric knee flexion contractions. However, two recent studies [31,34] reported no differences in peak EMG activity during maximal voluntary knee flexion between different hamstring lengths, achieved by altering hip angle. In the present study, lower EMG activity during modified variations of NHE was also observed for other muscles, suggesting that these were unloaded as well. The only exception was the gluteus maximus activity, which was relatively low during all variations of the NHE and did not significantly change with hip angle modification. Comparable (<20% MVC) level of activity during standard NHE for gluteus maximus were reported recently by Narouei et al. [35]. However, the activity of erector spine muscles was lower in their experiment (35–40% MVC) compared to our results (65% MVC in the standard NHE).

During the standard NHE, a higher peak in EMG activity of semitendinosus compared to biceps femoris was observed, with a difference of 6.5% (106.7 ± 15.5% MVC for semitendinosus and 99.7 ± 22.2% MVC for biceps femoris). Similar or even larger differnces in muscle activity between semitendinosus compared to biceps femoris during the standard NHE were reported by other researchers [16,36,37]. This variability in findings could be related to differences in normalization procedure of peak hamstring EMG (e.g. maximal isometric voluntary contraction or maximal sprinting) and to different methodological approaches to quantification of hamstring activity, namely functional MRI [37] and EMG [16]. Since biceps femoris is injured more often than semitendinosus, other exercises for hamstring conditioning should be considered to be included within a training regimen. For instance, supine leg curls and stiff-leg deadlifts were shown to target biceps femoris [29,38,39] more than the other hamstring muscles. It was also shown that lateral rotation of the tibia increases biceps femoris activity compared to semitendinosus during isometric knee flexion [40]. However, such adjustment is not possible for NHE, since the rotation of the tibia cannot be maintained when approaching knee extension.

Several limitations of the study should be acknowledged. In the present study, a familiarization session was not performed before the trials. While all participants had previous experience with resistance exercise and were instructed to perform two familiarization repetitions of each variation, we cannot rule out the presence of the learning effect. Although the variations of NHE were performed in a randomized order, such effect could nonetheless influence our outcomes. Furthermore, participants in our study were not able to maintain all of the instructed hip flexion positions during NHE. This way, the part of the experiment that was conducted to reveal the effects of different hip flexion positions merely showed the effects of instructions to the participants, not the effect of actual hip angles. Participants often flexed the lumbar spine instead of the hip, which could impose undesirable forces on the spine. Lastly, a large number of eccentric repetitions of the same muscle group in the single session could lead to a significant level of fatigue, which could affect peak torque or EMG activity during NHE variations and MVC procedures carried out at the end of the session. Considering all of the above, the results of this study need to be verified in an experiment that includes trained athletes with high level of NHE stregth.

## Conclusion

The presented modifications of NHE can be used for the purpose of individualization and optimization of strength and conditioning interventions, injury prevention and rehabilitation. It is likely for athletes to progress faster and more efficiently using suggested variations and appropriate progression. In particular, this study has demonstrated that an increase in the slope of the lower leg support allows more controlled descending throughout larger range of motion while reaching similar peak knee and hip torque as in the standard NHE. Individuals who are unable to perform the standard NHE through full range of motion will therefore probably benefit from adjusted slope support, before progressing towards the standard (i.e. horizontal) position of the lower leg. Performing NHE with an increased hip flexion angle may also be effective; however, athletes may change the positon of the spine as well as the hip when larger hip flexion angles are instructed to be maintained during NHE.
